# Embryo mix-up due to erroneous identification in the context of medically assisted reproduction

**DOI:** 10.1007/s10815-026-03875-5

**Published:** 2026-05-26

**Authors:** Francisca N. Amoah, Sarah Kehr, Susanne Müller, Markus B. Funk, Philipp Berg

**Affiliations:** 1 Division Safety of Biomedicines and Diagnostics, Paul Ehrlich Institut, Langen, Germany; 2 Division Hematology, Cell and Gene Therapy, Paul Ehrlich Institut, Langen, Germany

**Keywords:** IVF, Embryo transfer, Mismatch, Medical error, Quality management, Patient safety

## Abstract

The accurate identification of gametes, embryos, and patients is a fundamental requirement for the intended treatment in the context of assisted reproductive technology (ART). Embryo mix-ups are among the most serious errors in ART and are considered highly sensitive, which can impede error management and the identification of error sources. Here, we present five cases of embryo mix-ups that occurred at five different ART facilities. These reports highlight misidentification of patients and specimens as underlying factors in all five mix-up cases and emphasize the importance of facility-wide root cause analysis to improve process safety for patients and staff. Although human error was an important factor, the underlying conditions that make these critical errors possible must be identified to allow for effective risk mitigation. In all cases, a sequence of errors led to embryo mix-ups that could have been prevented by adherence to clear institutional guidelines and workflows regarding patient identification and verification of patient and sample identities prior to embryo transfer. It is important that operating guidelines are integrated into daily work routines and ART facilities have control systems in place to monitor critical steps and allow for the detection of error sources or faulty processes to prevent subsequent damage.

## Introduction

An increasing number of individuals and couples use assisted reproductive technologies (ART) to achieve pregnancy [1]. Accurate identification and traceability of gametes, embryos, and patients is a prerequisite for the correct treatment in the context of ART. The collection, processing, storage, and transfer of gametes and embryos involve complex clinical and technical steps with the potential risk of loss, mislabelling, and misidentification. Optimized processes and correct assignment are therefore crucial and subject to best-practice guidelines [2–4, 27]. For critical steps, double-checking by means of electronic or manual (four-eye principle) witnessing is strongly advised [2]. Errors in the traceability system that lead to an incorrect assignment of gametes or embryos and recipients are among the worst possible adverse events with devastating effects on those affected [5, 6]. Although such mix-ups are considered rare events and may remain undetected and hence unreported, isolated cases have been documented in several countries, demonstrating that this can occur across centers and regulatory systems [7–13].

Any type of gamete or embryo misidentification or mix-up qualifies as a serious adverse event according to the European Directive 2006/86/EC. In regulation (EU) 2024/1938, gametes and embryos are classified as reproductive substances of human origin (SoHO) in the context of medically assisted reproduction and a mix-up is defined as a serious adverse event subject to vigilance activities. Terminology may differ in different jurisdictions, with medicinal products of human origin (MPHO) or reproductive human cells, tissues, and cellular and tissue-based products (HCT/Ps) also used in this context. Importantly, a survey conducted by the European Society of Human Reproduction and Embryology (ESHRE) indicated considerable variability with regard to error management and reporting practices to national regulatory authorities as well as disclosure to patients [5]. While mix-up cases are believed to be related to human error, it is important that the systematic investigation of such errors or near-miss events focuses on the organizational context, such as workflows, standard operating procedures, and workload, rather than on individuals [14]. Adequate error management is key to achieving system-level improvements and further increasing control measures in the context of ART [5]. Identifying critical steps at which deviations occur and lead to the misdirection of gametes or embryos allows for a better understanding of underlying risks and subsequent risk minimization.

## Methods

Tissue establishments and medical care facilities that collect, process, or use human tissues or cells in the context of assisted reproduction are subject to vigilance regulations under the German Medicinal Products Act (Arzneimittelgesetz, AMG) and the German Transplantation Act (Transplantationsgesetz, TPG) [18], which align with the European legislation. There is a reporting obligation for suspected serious adverse reactions and serious adverse events. The Paul-Ehrlich-Institut, as the national regulatory authority, provides standardized reporting forms and captures all reports in a national database. A search of the database was conducted for reports relating to embryo mix-ups. All identified cases reporting embryo mix-up, misdirection or misidentification were included in the further evaluation. Sensitive information that could allow for the identification of individuals was not processed; instead, case numbers were assigned. Reports were manually assessed with regard to information on the circumstances of the mix-up, possible contributing factors, and consequences.

## Case series

Here, we present case reports in which transferred embryos and recipients did not match (Tables 1 and 2) and comment on contributing factors and risk mitigation. Between 2022 and 2024, five cases of embryo mix-up were reported from five different ART facilities in Germany. All cases were due to misidentification, either of the patient, the embryo, or the matching of both (Table 2). Patient identification (ID) failed in three cases (Cases 1–3) due to lack of confirmation of the patient’s identity (Table 2). In addition to the lack of clear identification of the recipient, tightly timed procedures, late arrival of the intended recipient, and communication difficulties were stated as additional factors. In one of the three cases, it was stated as unclear how miscommunication may have occurred. In a fourth case, a patient-embryo mismatch was reported although the patient had been correctly identified and the correct embryo had been prepared. However, embryos for several patients had been prepared and a wrong embryo was handed to the gynecologist. Since no comparison of the embryo ID with the recipient took place at this point, contrary to internal guidelines, the patient was transferred with the embryo of a different person scheduled for transfer on the same day (Case 4). In the fifth case, the mix-up occurred at the point of retrieving the embryo from the cryotank (Case 5). The identity of the retrieved embryo had not been double checked, as stated in the applicable standard operating procedure, and the error was also not noticed at the time of the embryo transfer. In total, seven embryos had been lost due to the error of misidentification.

**Table 1:**
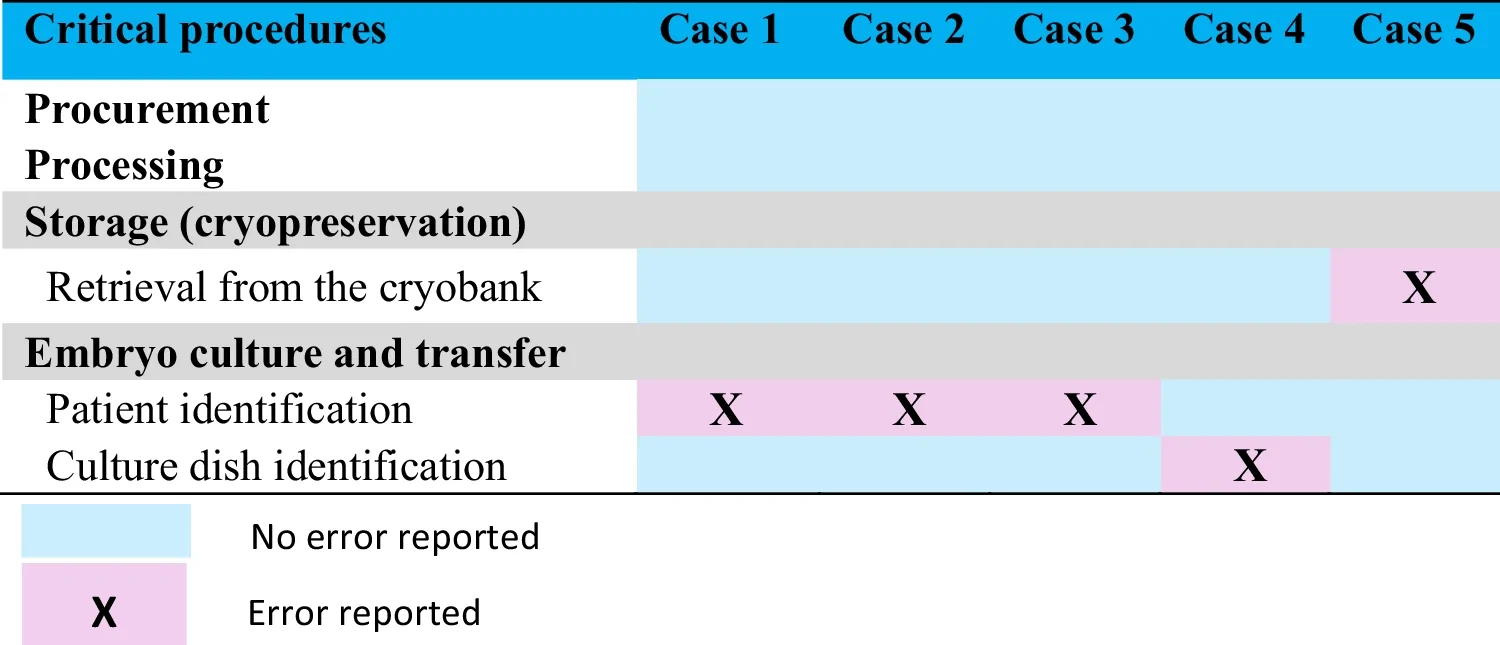
Overview of critical procedures and error occurrence in embryo mix-ups

**Table 2:** Summary of cases of embryo mix-up reported between 2022 and 2024

Case	Error description	Outcome	Contributing factor(s)	Potential preventive measures
1	Incorrect recipient followed the doctor’s call into the ET room; no ID verification; two embryos intended for a different recipient were transferred	Measures to prevent pregnancy initiated; no pregnancy occurred No embryo left for the intended recipient	Intended recipient was late for the appointment No double checking	• Patient self-identification (stating name, DOB) • Patient photo ID or EWS ID verification • Verification of patient and sample prior to ET • Double checking (2 HCP or EWS)
2	Incorrect recipient followed the doctor’s call into the ET room; no ID verification. The couple were referred to by the wrong name several times but raised no objection. The patient did not speak German fluently	Measures to prevent pregnancy initiated; pregnancy still occurred. The patient decided to not terminate the pregnancy	Intended recipient was late for the appointment Incorrect recipient arrived early Language barrier No double checking	
3	Patient verbally confirmed the incorrect name given by the embryologist	Measures to prevent pregnancy initiated; no pregnancy occurred	Miscommunication No double checking	
4	Recipient and specimen mismatch in ET room. Correct recipient ID, but the wrong specimen had been made available from multiple embryos to be transferred on that day	Measures to prevent pregnancy initiated; no pregnancy occurred	High workload No double checking	• Verification of patient and sample prior to ET • Double checking (2 HCP or EWS) • Working with material of only one patient at a time
5	The wrong sample was taken from the nitrogen tank. No comparison between recipient and specimen in transfer room	Pregnancy occurred. The couple terminated the pregnancy and were offered psychiatric assistance		• Verification of patient and sample prior to ET • Double checking (2 HCP or EWS)

*ET* embryo transfer, *DOB* date of birth, *ID* identification, *HCP* health care professional, *EWS* electronic witness system

In four out of five cases (80%), the error was noticed immediately after the transfer and measures were initiated to prevent pregnancy. Despite these measures, one woman became pregnant with the unmatched embryo and, after confirmation of the pregnancy, opted to continue the pregnancy and carry the genetically unrelated baby to term. In one case (20%), the mix-up was only discovered 2 months later, by which time a pregnancy had already occurred. After being informed, the couple decided to terminate the pregnancy with the intention to achieve a pregnancy with a genetically related embryo, and psychological trauma support was consulted. Overall, in two out of five cases (40%), women became pregnant with an unmatched embryo: one chose to terminate the pregnancy, while the other decided to carry the pregnancy to term, highlighting the range of possible outcomes.

In all reported cases, the embryo mix-up resulted in some form of medical intervention to prevent or terminate pregnancy, highlighting its classification as a serious adverse event.

It is noted that in cases of an embryo mix-up, there are always two patients or couples affected. In addition to the erroneous recipient, there is also the woman or couple for whom the embryo was originally intended. While most reports stated that all concerned patients were informed, in one case the intended recipient was informed about the loss of an embryo but not the underlying reasons. In one case, the couple whose embryo was transferred to another person had no embryo left.

None of the five concerned facilities had an electronic witnessing system (EWS) in place or adhered strictly to double-witnessing prior to embryo transfer. In two cases, a high workload on the day of transfer was mentioned and for the other three cases, no information on the volume of work was provided. To prevent the reoccurrence of such events, the reporting facilities revised standard operating procedures regarding patient and/or embryo identification, conducted additional staff training for involved personnel, and planned the implementation of an electronic witnessing system (EWS).

## Discussion

The reports highlighted misidentification of both patients and specimens as the underlying factor for the mix-ups. Although these cases can be classified as human error, the underlying conditions that make these critical errors possible must be identified to allow for risk mitigation. Adherence to clear institutional guidelines and workflows regarding patient identification as well as verification of both patient and sample identities prior to embryo transfer could have prevented these cases. It is important to note that operating guidelines must be practical and integrated into daily work routines, and compliance should be monitored additionally.

Ensuring consistently accurate patient identification has been perceived as a challenge in the healthcare sector for a long time [15] and errors are still a cause for avoidable patient morbidity and mortality, for example in the context of blood transfusion [e.g., 16]. Procedures related to ART can cause adverse reactions, such as the ovarian hyperstimulation syndrome (OHSS) or other complications including bleeding or infections [1, 17, 18]. However, the potential mix-up of biological samples is particularly devastating and this possibility is a reason for concern on the part of patients [19]. Therefore, the correct and unambiguous identification of a patient and her reproductive tissues is crucial in ART and has received particular attention in guidelines. The ESHRE guidelines for good practice in IVF laboratories explicitly stated that “Patients should be directly asked to give their own identifying information (at least full name and date of birth) before procurement or artificial insemination/embryo transfer” [2]. Communication failure results in a risk of patient misidentification and embryo mismatch [20]. Thus, reliance only on verbal confirmation of patient identity is not error-proof and stricter requirements can be an option, for example, by additional verification of the patient ID card against patient information. This might be particularly relevant in cases where language barriers exist, and no translator is involved in a procedural step. Confirming patient identity with the transferring doctor also fosters communication between the embryologist and the reproductive endocrinologist and minimizes the risk of an error [11], a step which was lacking in one of the reports, where the specimen information had not been matched to the patient file. Although three out of five mix-ups were reported in connection with incorrect patient identification, the likelihood of detecting errors related to patient misidentification is assumed to be higher than that of detecting mistakes related to sample or embryo misidentification.

To ensure the integrity of procedures in ART facilities and prevent mix-ups, the recommended best practice is a double-witnessing procedure at critical steps [2]. Double-checking can occur by means of electronic or manual (four-eye principle) witnessing but real-world practices may vary between centers and regulatory systems. The manual witnessing approach can be difficult to implement and error-prone, owing to the risk of independent redundancy, inattentional blindness, ambiguous accountability, and stress [21, 22]. Recommendations have been made to enhance human witnessing practices but the applicability may depend on the specific local circumstances [23]. Electronic witnessing systems (EWS) have been suggested as an alternative to manual witnessing, for example by barcoding or radiofrequency identification systems (RFID) tagging. These systems have shown substantial improvements in error rates for specimen identification if employed correctly [24]. Implementation of EWS in ART facilities has equally demonstrated success in improving identification, specimen traceability and preventing specimen mix-up [6, 24]. In addition, EWS allows for identifying error-prone steps as well as patterns, such as an increase of mismatches at a particular time of the day [6, 24], which could help to improve processes and further reduce sources of error. However, these studies also point to possible new sources of error caused by EWS procedures, which also require attention, and even the most reliable witnessing processes require a correct patient identification at the beginning.

A high workload was identified as a contributing factor to the omission of critical control steps in two of the cases reported here. Excessive workloads, time pressure, and understaffing in IVF laboratories can result in embryologist burnout and were highlighted as a safety concern contributing to witnessing errors [25, 26]. This underscores the need for systemic adjustments to support established best-practice procedures, reduce workload peaks, and ultimately ensure patient safety at all steps. The ESHRE and the American Society for Reproduction Medicine (ASRM) have published recommendations for staffing requirements depending on the number of treatments, complexity of the procedures, techniques and tasks undertaken within the laboratory [2, 27]. ART has evolved rapidly over the last decades and continues to evolve. It is therefore critical that ART staff receive regular training to ensure compliance with standard operating procedures and obtain up-to-date knowledge of the current scientific technologies. Notably, guidelines developed by ESHRE and the American Society for Reproductive Medicine provide recommendations for professional development of ART staff and the requirement for continual training. An open, non-punitive communication within facilities is crucial for learning from errors and near-misses to achieve system-level improvements [5, 28]. However, the sensitivity of mix-up cases makes transparent error management difficult in our experience. Even in affected facilities, such incidents may remain unknown to staff because they are treated as taboo, thereby limiting awareness of the issue. This case series aims to highlight contributing circumstances, encourage the reporting of errors, and emphasize the importance of facility-wide root cause analysis to improve process safety for patients and staff.

Beyond the emotional and potential legal implications, embryo mix-ups present ethical, social and legal challenges regarding the definition of parentage and associated rights and responsibilities. In one of the cases described here, a couple decided to carry a genetically unrelated embryo to term. The gestational mother giving birth to a child is recognized as the legal mother under German law (German Civil Code, BGB Sect. 1591). In other jurisdictions, embryo mix-ups have led to legal disputes and, in individual cases, genetic parents have been granted parental rights [13, 29]. Prince et al. [29] argue that parenthood extends beyond genetic ancestry and legal definitions, and that biological, social, and moral aspects must also be considered, and they emphasize the gestational relationship between child and mother.

## Conclusion

In conclusion, infertility is a stressful event that can be associated with anxiety and should not be compounded with preventable mismatch errors that further impede the chances of childbearing. For every procedure performed in the ART laboratory, several critical steps are required to ensure the correct identification of patients and their samples. Clear workflows should specify the identification method, unique identifiers, and a system with fail-safe mechanisms. These protocols could also include a checklist actively used for the implementation of each procedure. It should also indicate procedures in the event of an identity mismatch, including error handling and notification of the patient involved, as well as notification to competent authorities. The risk of human error can be reduced but not completely eliminated. Therefore, it is crucial that ART facilities have control systems in place to monitor critical steps and allow for the detection of error sources or faulty processes to prevent subsequent damage. The data collection shows that in all cases, it was not a single human error but a sequence of several errors that ultimately led to the embryo mix-up.

## Data Availability

The data underlying this article are available from the authors upon reasonable request but restrictions apply due to reasons of sensitivity.

## Declarations

### Disclaimer

The views expressed in this article are the personal views of the authors and may not be understood or quoted as being made on behalf of or reflecting the position of the agencies or organizations with which the authors are affiliated.
